# Genotoxic stress increases cytoplasmic mitochondrial DNA editing by human APOBEC3 mutator enzymes at a single cell level

**DOI:** 10.1038/s41598-019-39245-8

**Published:** 2019-02-28

**Authors:** Bianka Mussil, Rodolphe Suspène, Vincent Caval, Anne Durandy, Simon Wain-Hobson, Jean-Pierre Vartanian

**Affiliations:** 1 Molecular Retrovirology Unit, Institut Pasteur, CNRS, UMR 3569, 28 rue du Dr. Roux, F-75724 Paris cedex 15, France; 2 INSERM UMR 1163, The Human Lymphohematopoiesis Laboratory, Institut Imagine, 24 boulevard du Montparnasse, F-75015 Paris, France; 3 Present Address: Unit of Infection Models, German Primate Centre, Kellnerweg 4, D-37077 Goettingen, Germany

## Abstract

Human cells are stressed by numerous mechanisms that can lead to leakage of mitochondrial DNA (mtDNA) to the cytoplasm and ultimately apoptosis. This agonist DNA constitutes a danger to the cell and is counteracted by cytoplasmic DNases and APOBEC3 cytidine deamination of DNA. To investigate APOBEC3 editing of leaked mtDNA to the cytoplasm, we performed a PCR analysis of APOBEC3 edited cytoplasmic mtDNA (cymtDNA) at the single cell level for primary CD4^+^ T cells and the established P2 EBV blast cell line. Up to 17% of primary CD4^+^ T cells showed signs of APOBEC3 edited cymtDNA with ~50% of all mtDNA sequences showing signs of APOBEC3 editing – between 1500–5000 molecules. Although the P2 cell line showed a much lower frequency of stressed cells, the number of edited mtDNA molecules in such cells was of the same order. Addition of the genotoxic molecules, etoposide or actinomycin D increased the number of cells showing APOBEC3 edited cymtDNA to around 40%. These findings reveal a very dynamic image of the mitochondrial network, which changes considerably under stress. APOBEC3 deaminases are involved in the catabolism of mitochondrial DNA to circumvent chronic immune stimulation triggered by released mitochondrial DNA from damaged cells.

## Introduction

The APOBEC3 (A3) locus encodes a series of seven genes encoding six functional endogenous cytidine deaminases with substrate specificity for single stranded DNA (ssDNA)^1^. They leave DNA peppered with uracil residues. This process is referred to as genetic editing as it occurs post replication. A3 enzymes leave a telltale editing signature in DNA: most A3 enzymes preferentially edit a cytidine residue in the context of 5′TpC with the exception of A3G, which prefers 5′CpC dinucleotides^2–6^. The antiviral role of these A3 enzymes was initially highlighted by their impact on HIV and HBV replication^6–9^ and the fact that several *A3* genes can be up-regulated by interferon α^10–12^.

A3 can restrict the transposition of SINE and LINE retroelements^13,14^ and mitochondrial DNA (mtDNA) in cell lines and tissues, all of which show the A3 editing signature – 5′TpC and 5′CpC are preferentially deaminated^15^. For the latter, the target was cytoplasmic mtDNA (cymtDNA) in keeping with the observation that all A3 enzymes are unable to access the mitochondrial network^15^. In addition, A3A and to a lesser extent A3B, were shown to target chromosomal DNA non-specifically, leading to hypermutated DNA with up to 70% of cytidine residues deaminated^15,16^. A3A and A3B-induced editing occur predominantly on the lagging strand during DNA replication^17,18^. Interestingly, A3A and A3B can edit 5-methylcytidine (5MeC) residues in ssDNA which makes sense given that 5MeC is found exclusively in the nucleus^16,19–21^. Uracil bases in DNA are excised by uracil N-glycosidase (UNG) that initiates DNA damage responses, which can result in either DNA repair or catabolism.

Not surprisingly, high levels of A3A-induced DNA damage leads to the formation of double stranded DNA breaks (DSB), cell cycle arrest and apoptosis^22,23^. In conjunction with 5MeC deamination and DNA rearrangements, multiple 5′TpC-> 5′TpT mutations are among the principal hallmarks of many cancer genomes^16,24,25^. Recent genetic data coupled A3A editing to the development of breast, ovarian and hepatitis B virus associated liver cancer^26–29^.

Mitochondrial DNA resembles bacterial DNA in that it is unmethylated. When leaked to the cytoplasm, it can trigger cytoplasmic DNA sensor molecules, leading to inflammatory responses^30,31^. Recent work showed that mtDNA induces Toll-like receptor 9-mediated inflammatory responses in cardiomyocytes leading to myocarditis^32^. Similar mechanisms might play a role in other non-infectious chronic diseases or after severe bodily injury. While most effete mitochondria are phagocytosed, some mtDNA fragments clearly find their way to the cytoplasm, thereby acting as a danger signal^15,33^. A3 initiated catabolism of cymtDNA is in fact a mechanism for lowering the danger signal^33^. Indeed, if cytoplasmic DNA was not degraded, the stressed cell would be remorselessly driven to apoptosis. That A3-targeted cymtDNA is earmarked for catabolism as highlighted by the finding that PBMC DNA from *ung*^*−*/*−*^ patients showed higher levels of deaminated cymtDNA^15^.

Even a population of clonally derived cells shows heterogeneity in protein expression – for example, the number of molecules of any cell surface molecule can vary by two orders of magnitude. Given this, we hypothesized that intracellular cymtDNA sensing should vary considerably between individual cells depending on stress, leakage to the cytoplasm and the dynamics of cymtDNA catabolism. It is not possible to quantitate accurately cymtDNA and even less so for single cells. However, A3 editing of cymtDNA can be used as a marker of mtDNA release from the mitochondrial network and if analyzed on single cells would provide a singular insight into danger signaling. Here, we show that A3 catabolism of cymtDNA is highly variable among single cells and can involve up to 17% of peripheral blood CD4^+^ T cells. Genotoxic stress in *ung*^*−*/*−*^ cells can increase ~40 fold the number of cells showing A3-edited cymtDNA, which parallels the up-regulation of *A3A*, *A3F*, *A3G* and *A3H*. The quantities of edited cymtDNA are considerable revealing a very dynamic image of the mitochondrial network.

## Results

### Edited cymtDNA in single primary CD4^+^ T lymphocytes

Cytoplasmic mtDNA (cymtDNA) remains the most sensitive target to detect A3 editing^15^. As numerous studies have described substantial expression of all *A3* genes in leukocytes^12,34^, we purified CD4^+^ T lymphocytes from two healthy donors (D1 & D2). Ficoll-purified, non-stimulated CD4^+^ T lymphocytes were collected after overnight culture. Viable single cells were FACS sorted into 96-well PCR plates. As U-enriched A3 edited cymtDNA melts at a lower temperature than the parental DNA sequence, by using a restrictive denaturation temperature (Td), CG-> TA hyperedited cymtDNA could be selectively amplified. Approximately ~200 cells each were analyzed at a fixed Td of 85 °C in order to have a rapid readout of the mutation frequency of edited cymtDNA. As a signal differing by up to 1.7 °C could be sometimes detected across thermal cycler heating blocks^35^, a Td of 85 °C was chosen in order to be comfortably below the threshold of ~86.7 °C that allows recovery of hyperedited *MT-COI* (mitochondrial cytochrome c oxidase subunit I) DNA^15^.

**Figure 1:**
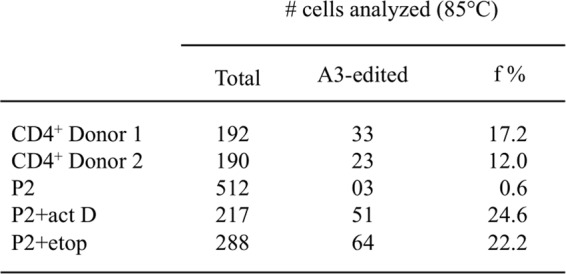
Frequencies of A3-edited cymtDNA in single cells. Frequencies of single cells harboring A3-edited cymtDNA using a fixed PCR denaturation temperature of 85 °C. Analysis was performed in single CD4^+^ T lymphocytes from 2 donors D1 and D2, ~17% and 12% of cells scored positive for APOBEC3 edited cymtDNA. Among the 512 P2 *ung*^*−*/*−*^ cells analyzed at 85 °C, 3 cells (0.6%) are positive. P2 *ung*^*−*/*−*^ cells were treated with 100 μM actinomycin D (act D) or etoposide (etop) for 16 hours. Treatment increased the proportion of cells showing evidence of cymtDNA editing to ~24.6% and 22.2% with actinomycin D or etoposide respectively. f: frequency, #: number.

**Figure 2:**
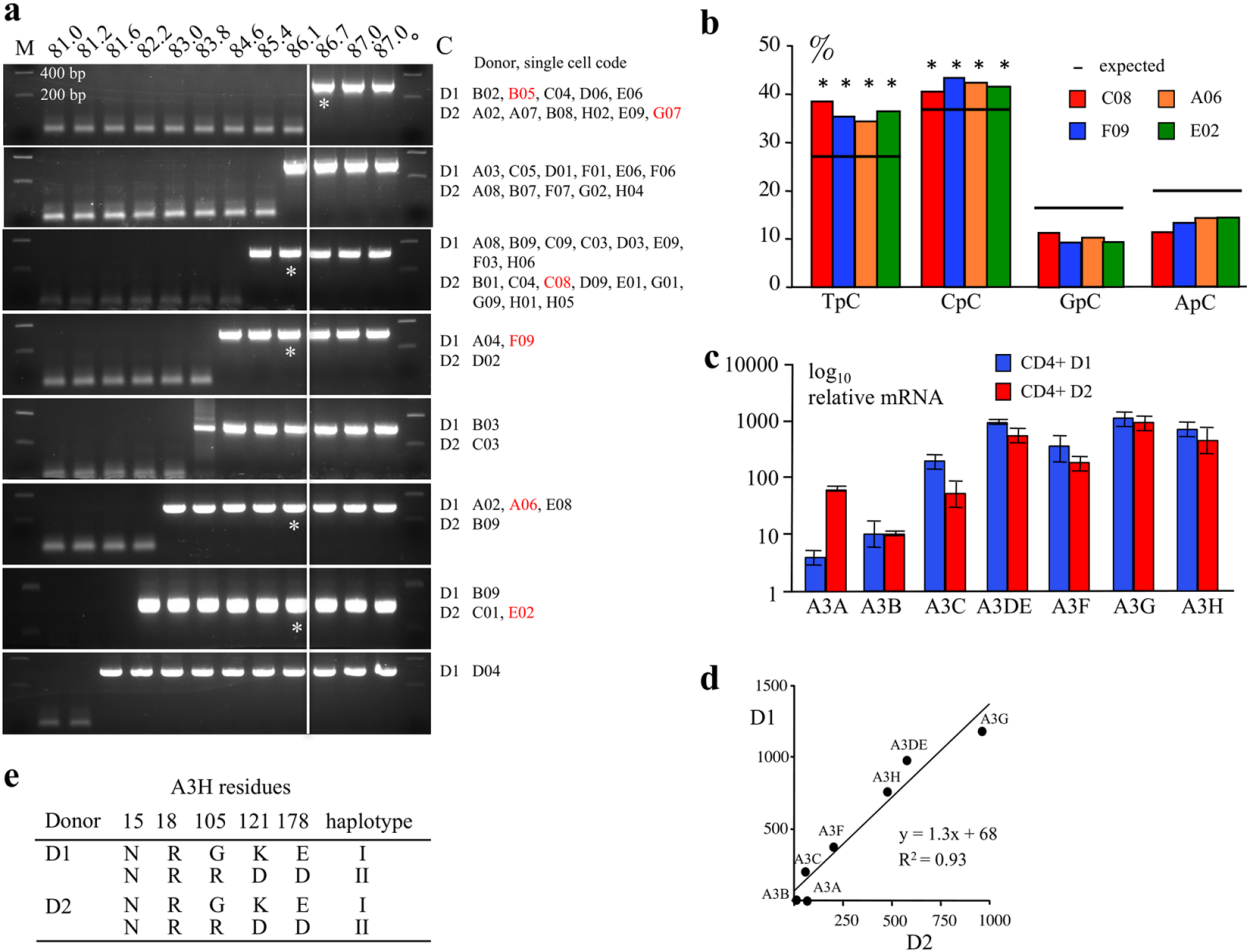
Editing of *MT-COI* in single CD4^+^ T lymphocytes from donor 1 and 2. (**a**) APOBEC3 edited cymtDNA was analyzed using a Td gradient across the heating block on first round PCR products. There were considerable differences in the extent of A3 editing as judged by the lowest Td at which edited DNA could be recovered. 3D-PCR recovered edited cytoplasmic *MT-COI* DNA down to 81.6–86.1 °C for 27 single cells from donor 1 (D1) and 25 single cells from donor 2 (D2). The Td just below the restriction temperature (86.7 °C) of unedited DNA (cell B05 and G07) were cloned and sequenced (Supplementary. Fig. S1). Cells F09, A06 from donor 1 (D1) and C08, E02 from donor 2 (D2) in red obtained at 86.1 °C were analyzed in detail. The white line indicates the threshold between edited and unedited 3D-PCR products in terms of the denaturation temperature. Cells B05 and G07 showed no editing of cymtDNA and were used as control. Asterisks refer to the samples cloned and sequenced. M: molecular weight markers. (**b**) Dinucleotide context of *MT-COI* DNA region minus strand DNA obtained from cells C08, F09, A06 and E02. The horizontal bar represents the expected values of dinucleotide composition (expected). Chi-square test indicates dinucleotide frequencies that significantly deviate from the expected values (*p < 0.05). (**c**) TaqMan analysis of *A3* transcriptome form positively selected but unstimulated CD4^+^ T cells from donor 1 (D1) and 2 (D2). Data in triplicate were normalized to the expression levels of *RPL13A* housekeeping reference genes. (**d**) Correlation between the A3 gene transcription levels for the two donor samples, D1 and D2. All seven *A3* genes were expressed with the relative mRNA levels being well correlated between the two donors (**e**) APOBEC3H haplotypes of the 2 donors D1 and D2. Genetic analysis showed mixed haplotypes I/II.

With a cut-off used of Td = 85 °C, ~17% and 12% of cells from donors D1 and D2 scored positive for APOBEC3 edited cymtDNA (Fig. 1). To refine the analysis, APOBEC3 edited cymtDNA was analyzed using a Td gradient across the heating block, a technique called 3D-PCR (differential DNA denaturation PCR)^36^ on first round PCR products for 27 and 25 single cells from donors D1 and D2 respectively (Fig. 2a). As can be seen, there were considerable differences in the extent of A3 editing as judged by the lowest Td at which edited DNA could be recovered. To explore A3 editing at the molecular level, 3D-PCR products recovered at 86.1 °C, the first Td just below the restriction temperature of unedited DNA were cloned and sequenced for two cells showing signs of cymtDNA editing from each donor (D1: A06 & F09; D2: C08 & E02; Fig. 2a red, Supplementary Fig. S1) as well as two cells where there was no editing (Td = 86.7 °C; D1: B05; D2: G07; Fig. 2a red). There was massive A3 editing of cymtDNA for the first four cells with overall C-> T mutation frequencies around 12% compared to 0.6% for the control cells B05 and G07. The mutation matrices of sequences recovered at 86.1 °C confirmed that the cymtDNA was indeed edited (Supplementary Fig. S1). The 5′ dinucleotide context associated with editing was strongly in favor of 5′TpC and 5′CpC, which is typical for A3 deaminases (Fig. 2b). Hence with a cut-off of 86.1 °C ~81% (22/27, Fig. 2a) and ~76% (19/25, Fig. 2a) of cells from donors D1 and D2 scored positive for APOBEC3 edited cymtDNA (Fig. 2a).

To assess the relative expression levels of *A3* genes, a TaqMan transcriptional study was made on bulk CD4^+^ T cells of the seven *A3* genes using *RPL13A* as reference (Fig. 2c). All seven *A3* genes were expressed with the relative mRNA levels being well correlated between the two donors (Fig. 2d). As 5 functional enzymes were expressed (A3A, A3C, A3F, A3G and A3H), it is possible that all are involved. A3DE is an inactive protein although it can modulate A3F and A3G in a negative manner^37,38^ while human A3B does not edit cymtDNA presumably because it is translocated too rapidly to the nucleus^15,16^. For *A3H*, there are seven haplotypes not all of which encode stable enzymes. Haplotype II encodes the most active form of A3H, while haplotype I is much less active^39,40^. Genetic analysis showed mixed haplotypes I/II indicating that A3H haplotype II could also be participating in editing of cymtDNA (Fig. 2e).

**Figure 3:**
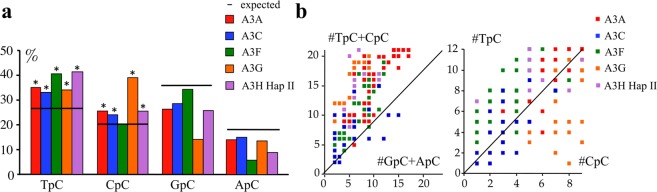
Dinucleotide context and clonal analysis of *MT-COI* editing in QT6 quail cells. (**a**) Bulk dinucleotide context of quail *MT-COI* DNA edited by A3A, A3C, A3F, A3G and A3H Hap II cytidine deaminases. The horizontal bar represents the expected values of dinucleotide composition (expected). Chi-square test indicates dinucleotide frequencies that significantly deviate from expected values (*p < 0.05). (**b**) Clonal analysis of *MT-COI* editing for A3A, A3C, A3F, A3G and A3H Hap II cytidine deaminases. The number (#) of TpC + CpC vs. GpC + ApC targets edited per sequence are computed and represented on the y and x axes respectively (left), and clonal analysis using TpC vs. CpC (right). The number (#) of TpC + CpC vs. GpC + ApC targets edited per sequence and represented on the y and x axes respectively (left), and clonal analysis using TpC vs. CpC (right). Some dots overlap due to the identical number of APOBEC3-edited sequences.

**Figure 4:**
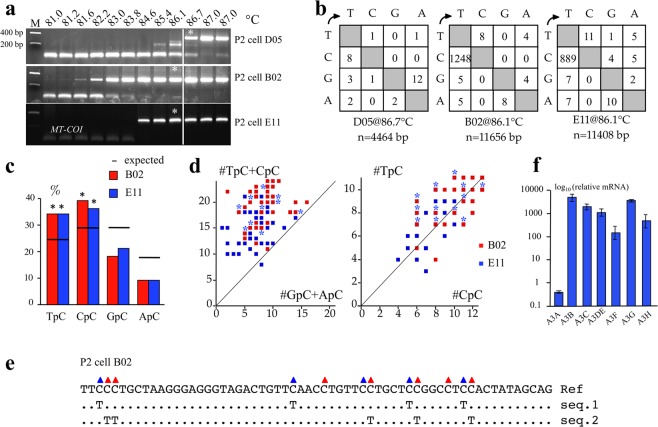
*MT-COI* editing in P2 single cell. (**a**) 3D-PCR recovered edited *MT-COI* DNA down to 81.2 °C for single cells B02 and down to 84.6 °C for single cell E11. Cell D05 did not harbor edited mtDNA and was used as a control. The white line indicates the threshold between edited and unedited 3D-PCR products in terms of the denaturation temperature. (**b**) Mutation matrices for hyperedited *MT-COI* DNA sequences from cells D05, B02 and E11 derived from cloned 3D-PCR obtained at 86.7 °C and 86.1 °C. The numbers below the matrices (n) indicate the number of nucleotides analysed. (**c**) Dinucleotide context of *MT-COI* DNA region minus strand DNA obtained in cell B02 and E11. The horizontal bar represents the expected values of dinucleotide composition (expected). Chi-square test indicates dinucleotide frequencies that significantly deviate from expected values (*p < 0.05). (**d**) Clonal analysis of *MT-COI* editing for individual edited sequences from cell B02 and E11. Blue asterisks represent the overlapping sequences between cell B02 and E11. The number (#) of TpC + CpC vs. GpC + ApC targets edited per sequence are computed and represented on the y and x axes respectively (left), and clonal analysis using TpC vs. CpC (right). (**e**) Edited *MT-COI* DNA from P2 cell B02 at a single cell level in two different dinucleotide contexts, 5′TpC in blue triangle (seq1) and 5′CpC in red triangle (seq2). (**f**) TaqMan analysis *A3* transcriptome of bulk P2 cells. Data in triplicate were normalized to the expression levels of *RPL13A* housekeeping reference genes.

To get a better idea as to which human A3 enzymes were involved, quail QT6 cells were transfected by *A3* expression plasmids and the equivalent region of quail mtDNA analyzed (human, 248 bp, 25% T, 33% C, 18% G, 24% A, versus quail, 245 bp, 26% T, 33% C, 18% G, 23% A). As the quail genome does not encode an *A3* gene, there is no endogenous background editing^3^. A3A, A3C, A3F, A3G and A3H could all access quail cymtDNA as previously reported^15^. However, as A3H haplotype I is poorly expressed in QT6 as well as in HeLa cells (not shown), we performed editing and dinucleotide contexts analyses only with A3H haplotype II (Fig. 3). Comparisons of the dinucleotide context with those from bulk P2 single cells (an EBV transformed B-cell line obtained from a *ung*-deficient human patient)^41^ (Figs 3a and 4c), as well as single molecule analyses, which show the number of mutations per sequence according to the dinucleotide context (Figs 3b and 4d), indicate that A3A, A3C, A3F, A3G and A3H are all plausible candidates.

### Editing of cymtDNA in *ung*^*−*/*−*^ cell line

Detection of A3 editing is rate limited by the highly efficient enzyme UNG, whether it be nuclear, mitochondrial or viral DNA^15^. Accordingly, analysis of cymtDNA in EBV transformed B cells from rare patients with *ung*^*−*/*−*^ deficiency^15,41^ might show even greater proportions of edited DNA. Viable P2 *ung*^*−*/*−*^ cells were FACS sorted into 96 well PCR plates. Over 500 cells were analyzed by the PCR/3D-PCR procedure described above^15,36^. Among the 512 P2 cells analyzed at a fixed Td of 85 °C, only three (0.6%) proved positive which was a little surprising given the *ung*^*−*/*−*^ background (Fig. 1). First round *MT-COI* PCR products from cells B02 and E11, as well as those from a negative cell (D05) were then analyzed by 3D-PCR using a gradient of denaturation temperatures (Fig. 4a). As can be seen, *MT-COI* DNA was recovered down to Td 81.2 °C and 84.6 °C for cells B02 and E11. By contrast, no mtDNA was recovered below 86.7 °C for cell D05, the lowest temperature at which normal *MT-COI* DNA can be amplified. Cloning and sequencing of 3D-PCR products identified by an asterisk (Fig. 4a) showed massive editing of cymtDNA, ~17–24 fold greater (number of CG-> TA/total nucleotides) than the background (cell D05, Fig. 4b). Again, the dinucleotide context associated with cytidine editing showed a strong preference for 5′TpC and 5′CpC (Fig. 4c,d). Single molecule analysis showed that most were edited at 5′TpC and 5′CpC sites, even though locally some regions were exclusively edited at 5′TpC or 5′CpC sites, consistent with a degree of processivity typical of these enzymes (Fig. 4e). An *A3* transcriptome analysis on bulk P2 cells showed good expression of 6 *A3* genes. *A3A* levels are very low as is typical for established cell lines (Fig. 4f). As for the human donor CD4^+^ T cells, the likely contributions to editing are from A3C, A3F, A3G and A3H. This suggests that the low level of P2 cells harboring A3 edited cymtDNA probably reflects low levels of release of mtDNA into the cytoplasm rather than an absence of A3 enzymes in the cytoplasm. This is consistent with the fact that cymtDNA is a danger signal for the cell and is eliminated by a pathway involving the APOBEC3/UNG/APE enzymes^33^. Release of mtDNA within the cytosol along with the lack of UNG are probably deleterious for the cell.

### Genotoxic stress induced APOBEC3 expression and apoptosis

**Figure 5:**
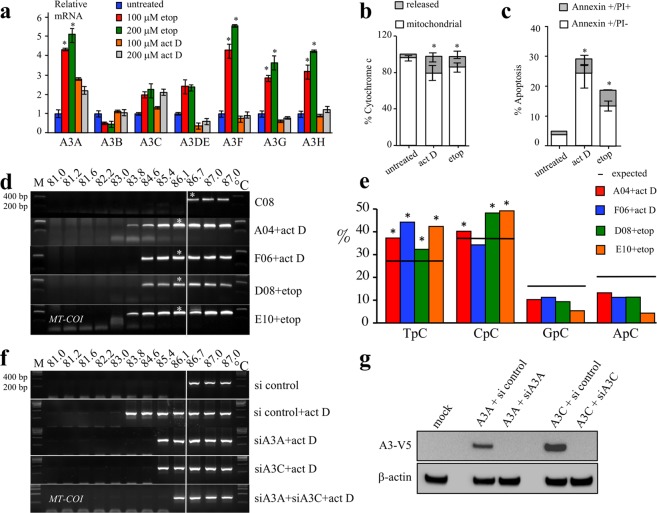
Etoposide and actinomycin D induced *A3* expression and *MT-COI* editing in P2 cells. (**a**) Transcription profiling of A3A-A3H in etoposide or actinomycin D treated-P2 cells. Data in triplicate were normalized to the expression levels of *RPL13A* housekeeping reference genes and to untreated P2 cells to facilitate comparison (*p < 0.05). (**b**,**c**) FACS analysis of cytochrome c release (left) and apoptosis (right) in P2 cells treated with 100 µM of actinomycin D or 100 µM etoposide after 16 hours. Annexin V scored early apoptosis and propidium iodide (PI) late apoptosis/necrosis. Means and SEM are given for three independent experiments (*p < 0.05). (**d**) 3D-PCR recovered edited *MT-COI* DNA down to 83.8–84.6 °C for P2 single cells A04 and F06 treated with actinomycin D and for P2 single cells and D08 and E10 treated with etoposide. Cell C08 served as unedited control. (**e**) Dinucleotide context for A3-edited *MT-COI* DNA. The horizontal bar represents the expected values of dinucleotide composition (expected). Chi-square test analysis indicates dinucleotide frequencies that significantly deviate from the expected values (*p < 0.05). (**f**) P2 cells were treated with 100 µM actinomycin D and transfected with 1μg of A3A and/or A3C siRNA knockdown. The white line indicates the threshold between edited and unedited 3D-PCR products in terms of the denaturation temperature. (**g**) Efficiency of A3A and A3C siRNAs (experiment was performed in triplicate).

The low frequency of P2 cells harboring hyperedited cymtDNA provides a low background for testing *A3* induction to stress. Given that *A3* genes can be induced by inflammatory cytokines such as type I and II interferons, TNFα as well as PMA^10,12,34,42,43^, we explored the impact of other forms of stress, notably genotoxic stress on *A3* gene expression. Etoposide is a topoisomerase II inhibitor, while actinomycin D binds to DNA and inhibits transcription initiation, both leading to apoptosis^23^. An *A3* transcriptome analysis showed up-regulation of *A3A, A3F, A3G* and *A3H* for etoposide and a slight increase of A3A and A3C after actinomycin D treatment using ~2-fold increase as cut off (Fig. 5a). Treatment by these molecules resulted in cytochrome c release and apoptosis at 16 hours (Fig. 5b,c).

After treatment with 100 μM etoposide or actinomycin D for 16 hours, viable P2 cells were individually FACS sorted into 96-well PCR plates and the proportion of cells harboring hyperedited cymtDNA at Td = 85 °C was determined as above. Treatment increased the proportion of cells showing evidence of cymtDNA editing ~40 fold to ~23% (Fig. 1). First round PCR products from two cells treated with 100 µM actinomycin D (A04 and F06) or 100 µM etoposide (D08 and E10) were analyzed in detail as described above (Fig. 5d) and compared to the untreated C08 cell. PCR products obtained at 86.1 °C were cloned and sequenced. CymtDNA was heavily hyperedited (Supplementary Fig. S2), with the dinucleotide context in favor of 5′TpC and 5′CpC (Fig. 5e). As etoposide activates *A3A*, *A3F*, *A3G* and *A3H* (A3DE is not functionally active)^37^, one or all of these enzymes could be involved in catabolizing cymtDNA. By contrast, actinomycin D up-regulates weakly only *A3A* and *A3C* genes (Fig. 5a). To demonstrate that cymtDNA is specifically edited by A3 molecules, P2 cells were transfected with A3A and/or A3C siRNAs and then treated with 100 μM actinomycin D. As expected and visualized in Fig. 5f, we detected less hyperedited cymtDNA in presence of A3A and/or A3C siRNAs. When compared to si control, the additional PCR product obtained at 86.1 °C in presence of A3A + A3C siRNAs suggests that A3 cytidine deaminases other than A3A and A3C could be also involved in cymtDNA editing. Efficiency of A3A and A3C siRNAs were confirmed by Western blotting (Fig. 5g).

### Single cell hypoedited cymtDNA

**Figure 6:**
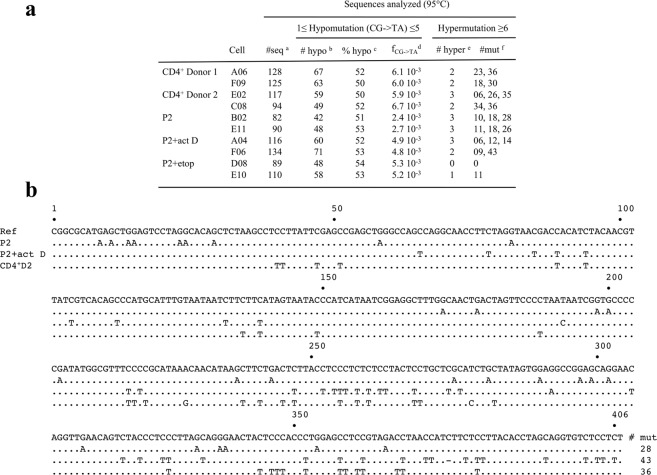
Abundant A3-edited cymtDNA in single cells. (**a**) Number of hypo- and hypermutated mtDNA sequences obtained using a PCR denaturation temperature of 95 °C; ^a^number of analyzed sequences; ^b^number of hypomutated sequences; ^c^proportion of hypoedited sequences per cell; ^d^mean CG-> TA mutation frequencies for hypoedited sequences, ^e^number (#) of hyperedited mtDNA sequences identified; ^f^number (#) of C-> T or G-> A transitions per mtDNA sequence. (**b**) A selection of hypermutated *MT-COI* sequences in presence or absence of 100 μM actinomycin D in P2 cells and in purified CD4^+^ from patient D2. Ref corresponds to the *MT-COI* reference sequence. Only differences are shown. To the right are the number mutations per sequence edited.

As 3D-PCR preferentially recovers heavily as opposed to lightly deaminated DNA, it is not a very quantitative technique^36^, we turned to deep sequencing to identify lightly deaminated cymtDNA. As it is not possible to purify cymtDNA from single cells, total mtDNA was sequenced. First round *MT-COI* PCR DNA recovered at 95 °C from all the single cells showing signs of hyperediting described in Figs 2–5, were cloned and sequenced. As a working definition, any sequence with ≤5 monotonous CG-> TA substitutions was considered to be hypomutated, while a hypermutated sequence was considered to harbor ≥6 such mutations. Sequences with non-CG-> TA substitutions were ignored. All 10 cells analyzed harbored approximately 50% of hypomutated mtDNA, the mutation frequencies per cell being 1–4 mutations per cymtDNA fragment or ~2–7 10^–3^ per base (10^3^ times higher than the Taq polymerase error)^44^, with the C-> T mutations located within the canonical 5′TpC dinucleotide. Hypermutated sequences were found at frequencies around 2% in samples obtained at 95 °C (Fig. 6a,b). The uniformity in the percentage of hypomutation is striking given the different genetic backgrounds and experimental systems. The frequency of hypomutated mtDNA detected is probably underestimated for two reasons: (1) the *MT-COI* DNA fragment sequenced is 248 bp long. It represents ~1/67 of mtDNA. As the *MT-CYTB* cymtDNA is edited in a similar manner^33^, absolute numbers of edited cymtDNA per cell are considerably greater; (2) UNG is rate limiting in the detection of A3 edited DNA^15^.

### Sepsis and circulating edited mtDNA

**Figure 7:**
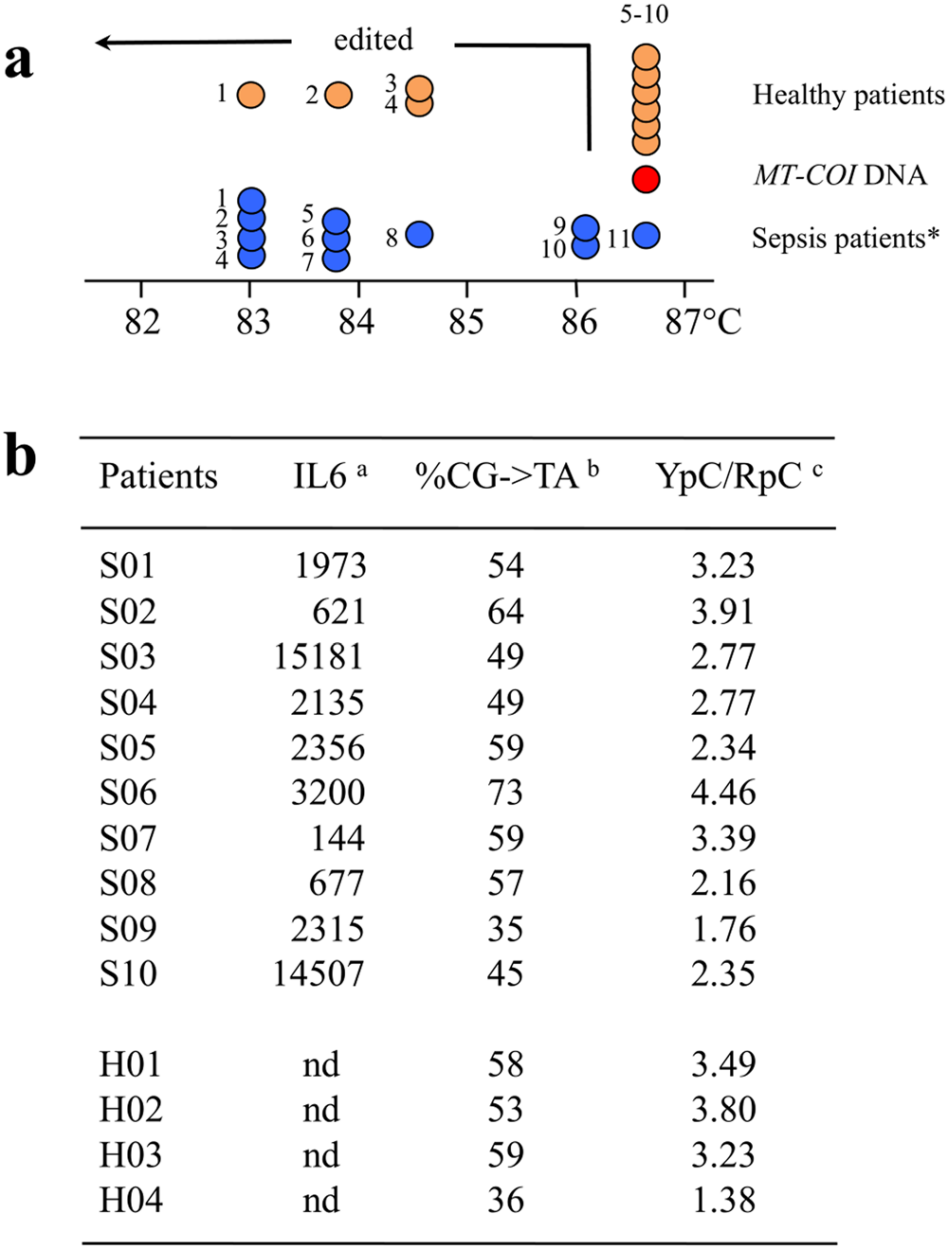
*MT-COI* editing in healthy and sepsis patients. (**a**) Schematic representing the denaturation temperature of the last positive 3D-PCR amplification for *MT-COI* DNA derived from the serum of sepsis and healthy patients. Red circle indicates a molecular *COI-MT* DNA clone of the reference sequence, blue circles represent serum samples from sepsis patients and orange circles represent serum samples from healthy patients. *Chi-square test indicates that mtDNA editing in sepsis patients significantly deviate from the healthy patients (p = 0.028; p < 0.05). (**b**) Sequence analysis of hyperedited *MT-COI* sequences obtained from 10 sepsis patients (S) and 4 healthy controls (H). ^a^Amount of serum IL6 (pg/mole), ^b^percentage of CG-> TA edits, ^c^YpC/RpC was calculated as follows: ((TpC + CpC)/(GpC + ApC)) observed/((TpC + CpC)/(GpC + ApC)) expected. A value > 1 is indicative of A3 cytidine deamination in TpC + CpC dinucleotide context. nd: not determined.

In view of an experimental link between cell stress and cytoplasmic mtDNA editing, we sought a link between stress and DNA editing in a more natural setting. Sepsis is generally characterized by systemic inflammation due to microbial invasion of the bloodstream^45^. As naked mtDNA can be found in serum or plasma, we extracted total DNA from the serum of 11 patients with sepsis^46^ compared to 10 healthy donors (Fig. 7a). *MT-COI* DNA was recovered by the same nested PCR/3D-PCR approach described above. *MT-COI* DNA was recovered at denaturation temperatures as low as 83.1 °C from 10/11 (~90%) sepsis samples tested (Fig. 7a). By contrast 4/10 healthy serum controls showed signs of editing (Fig. 7a). Molecular cloning and sequencing confirmed that they represented A3 edited *MT-COI* DNA with the classic 5′TpC and 5′CpC editing bias (Fig. 7b). Although no correlation was found between the degree of editing and serum levels of the pro-inflammatory cytokine IL6, as the proportion of positive samples from patients with sepsis was greater than control findings, our results could reflect the highly inflammatory microenvironment and release of necrotic cells into the periphery.

## Discussion

The A3 cytidine deaminases are particularly well expressed in hematopoietic cells^12,47,48^. They can edit cymtDNA, which is consistent with their cytoplasmic or nucleo-cytoplasmic localization; only A3B failed to do so, presumably because it is rapidly translocated to the nucleus^15^. The fraction of cells harboring A3-hyperedited cymtDNA in primary CD4^+^ T cells was ~12-17%, while in P2 EBV blasts, it could be increased ~40 fold by genotoxic stress (Fig. 1). Although the primary CD4^+^ T cells analyzed here were not linked to a functional subtype, they probably reflect cells downsizing their mitochondrial networks following the transition from proliferation to memory or resting cells. Alternatively, they could also reflect spillover into the blood of stressed cells following encounters with pathogens at a variety of sites throughout the body. This could represent a population of different cells rather than one cell subset with a precise phenotype. Moreover, it has been demonstrated that naïve (CD45RA^+^) and memory (CD45RO^+^) CD4^+^ T cells isolated from donors showed no differences between IFN α induction of mRNA of *A3A* to *A3H*, suggesting that probably mtDNA editing in naïve and memory CD4^+^ T cells would be identical^12^.

Hypermutated mitochondrial DNA can be recovered by a technique called 3D-PCR, which stands for differential DNA denaturation PCR^36^. This method exploits the fact that edited DNA is richer in AT compared with the reference. Modulation of the PCR denaturation temperature allows selective amplification of AT-rich DNA, sometimes by up to 10^4^-fold^49^. We can note that the data presented in Fig. 1 were performed by using a thermal cycler with a fixed denaturation temperature of 85 °C. Indeed, we used a fixed temperature to have a faster readout for analyzing the mutation frequency of edited cymtDNA for screening a large number of cells. The mutation frequency calculated for CD4^+^ T-cells in both D1 and D2 donors were ~12–17%. In Fig. 2a, as the Td cut-off of the 3D-PCR used is 86.1 °C, it seems that the mutation frequency (~81% and ~76% for donors D1 and D2 respectively) of edited cymtDNA could be different from the previous experiment performed at 85 °C due to technical discrepancies. Hence, application a Td of 85 °C (Fig. 1) will inevitably underestimated the number of edited cymtDNA molecules but will be *in fine* similar to the mutation frequency obtained in Fig. 2a with a cut-off of 84.6 °C.

Although the number of hyperedited cymtDNA molecules per cell was ~2%, range 0–4% (Fig. 6a), the number of hypoedited cymtDNA molecules was much greater, of the order of 50% (Fig. 6a). The mitochondrial network varies considerably between resting and proliferating cells and the number of mtDNA genomes varies accordingly, ranging from 3.10^3^–10^4^ per cell. Using these numbers, it is possible to calculate the number of A3-*hyperedited* cymtDNA molecules per cell which is of the order of (3.10^3^–10^4^) × 2% = 60–200 per cell at any moment. Likewise, the proportion of *hypoedited* sequences (1–5 CG-> TA mutations) is (3.10^3^–10^4^) × 50% = 1500–5000 copies per cell. These are phenomenal numbers and highlight a highly dynamic mitochondrial network in a substantial proportion of primary cells. The numbers show that not all mtDNA is catabolized within autophagosomes; a substantial proportion is catabolized in the cytoplasm by exonucleases. This follows from the fact that all A3 enzymes edit only ssDNA.

As up to 17% of unstimulated primary CD4^+^ T cells from healthy blood donors harbor hypermutated cymtDNA, what does this tell us? The fraction is too large to reflect proliferating effector cells in the periphery or reflect an antigen or pathogen specific population of cells. However, it could reflect activated cells that have recently come out of proliferation.

The findings tie in well with the massive egress of mtDNA to the cytosol after a mitochondrial genetic lesion, as well as infection stress following herpesvirus infection^50^. In this report, the signaling pathway was the DNA sensor cGAS that promoted STING-IRF3 dependent signaling resulting in IFN production. However these observations were made in the mouse, which unusually for mammals, encodes a single *A3* gene encoding a so-called Z2Z3 double domain A3 enzyme that is without equal in humans. Although different DNA sensors probably overlap and converge on induction of IFN and a vast array of downstream effector molecules^51^, catabolism of the DNA agonist in the mouse probably proceeds by a different mechanism for we were unable to detect cytidine deaminated mtDNA in primary tissues^15^.

Cytidine deamination of ssDNA is rapidly followed by removal of uracil moieties by the highly efficient enzyme UNG, followed by cleavage of the ssDNA by apurinic/apyrimidinic endonucleases such as APE1 and 2. Together the three are equivalent to a cytidine specific endonuclease. The fact that A3-edited mtDNA is found in so many situations - fresh donor PBMCs^15^, numerous cell lines^15^, etoposide and actinomycin D-treated P2 cells, while an orthologous A3A enzyme is conserved across 150 MYr of evolution - suggests that the cytoplasm regularly harbors ssDNA fragments requiring catabolism. In this context, it is interesting that Aicardi-Goutières patients with a *TREX1* lesion gene show signs of chronic inflammation^52,53^. Presumably, A3 cytidine deaminases and TREX1 act in concert to catabolize cytoplasmic DNA, whatever its origin. The higher frequency of edited *MT-COI* DNA present in the serum of sepsis patients (10/11, Fig. 7a) compared to healthy patients (4/10, Fig. 7a) suggests that the proportion increases with cell stress and/or inflammation, although there was no correlation with serum IL6 levels (Fig. 7b).

Is there any function left in hyperedited cymtDNA molecules? A3 editing generates U rich cytoplasmic mtDNA that doesn’t reanneal well and thus unable to drive internal DNA danger signalling^33^. So while the genetic information is totally lost, there is a selective advantage to APOBEC/UNG/APE catabolism of cytoplasmic DNA. The parallel with ADAR-1L adenosine editing of dsRNA is striking^54,55^.

## Methods

### Reagents and Plasmids

Etoposide was from Sigma and actinomycin D from Millipore. The V5-tagged A3A, A3C, A3F, A3G and A3H cDNAs in the pcDNA3.1D/V5-His-TOPO expression vector (Invitrogen) have been described^15^. All constructs were grown in *E. coli* DH5α. A3C siRNA (HSS120676, HSS120678, HSS178439), A3A siRNA (HSS153372, HSS153373, HSS153374) and siRNA negative control are from Thermoscientific.

### Cell culture and transfection

The Japanese quail muscle fibroblast cell line QT6 (ATCC^®^ CRL-1708^™^) was maintained as described^15^. For transfection, 7 × 10^5^ QT6 cells were seeded in six-well tissue culture plates and incubated for 24 hours. Transfections were performed using jetPRIME (Polyplus transfection). At 48 hours, DNA was extracted using the MasterPure Complete DNA and RNA purification kit (Epicentre Biotechnologies). Human P2 cells, an EBV transformed B-cell line obtained from an *ung*-deficient human patient^41^ were maintained as described^15^. Apoptosis was induced by incubating P2 cells with 100 µM actinomycin D or 100 µM etoposide for 16 hours. Cells were collected and used for DNA extraction.

### Patient samples and CD4^+^ T cell isolation

Blood was obtained from anonymous healthy donors (Authorisation IP: HS2004-3165 and HS2012-24917) and approved by the Comité Consultatif National d’Ethique (CCNE) de la Direction Générale et d’instances éthique et déontologique de l’Institut Pasteur. Federalwide Assurance (FWA) for the Protection of Human Subjects is FWA00003327 (N° IRB: 00006966). The anonymous healthy donors provided their written informed consent to participate in this study. In the context of the “Arrêté du 5 avril 2016 fixant les critères de sélection des donneurs de sang, Annexe IV”, we genotyped the APOBEC3H in CD4^+^ T lymphocytes cells from these healthy donors. Peripheral blood mononuclear cells (PBMCs) were isolated by Ficoll gradient (Eurobio). Isolation of CD4^+^ T lymphocytes was performed by incubation with antibody-coated magnetic beads (Miltenyi Biotec). Their purity of CD4^+^ T lymphocytes was above 90%, as checked by flow cytometry (FACSCalibur, Becton Dickinson). Two million CD4^+^ T lymphocytes were seeded in 24-well plates, cultured overnight and subsequently applied for single cell sorting. The sepsis patients have been described^46^ and the protocol has been approved by the ethical committee of Pitié-Salpétrière Hospital, Paris (NCT00698919). All methods were performed in accordance with the relevant guidelines and regulations. Informed consent was obtained from all participants and their legal guardians. Serum from healthy volunteers were obtained from sampling to distribution through the NSF 96–900 certified ICAReB platform (BB-0033-00062/ICAReB platform/Institut Pasteur, Paris, France/BBMRI AO203/1 distribution/access: 2016, May 19th, [BIORESOURCE]). Samples were obtained after informed consent under the Diagmicoll protocol approved by the Committee of Protection of Persons, Ile de France-1 (No 2010-dec-12483).

### Single cell sorting

P2 cells were collected 16 hours post-treatment with the apoptosis inducing drugs actinomycin and etoposide. In parallel, untreated P2 cells were harvested. CD4^+^ T lymphocytes were collected after overnight culture. All cells were resuspended in 500 µl PBS and viable single cells as defined by gating, were sorted into 96-well PCR plates (ABgene, Thermo Scientific) using the MoFlo (Beckman Coulter). Plates were immediately sealed with PCR adhesive film (Thermo Scientific), stored at −20 °C and analyzed by PCR without DNA extraction.

### Mitochondrial cytochrome c release

At 16 hours post-treatment with the apoptosis inducing drugs actinomycin and etoposide, P2 cells were investigated for cytochrome c release using the FlowCellect Cytochrome c Kit from Millipore, following the manufacturer’s instructions. Cells were analyzed with - using Cell Quest Pro (BD Biosciences) or FlowJo software (Tree Star, Inc., version 8.7.1). For each sample 10,000 cells were counted.

### FACS analysis for apoptosis

Annexin V possesses high affinity for the phospholipid phosphatidylserine (PS), thereby identifying cells undergoing apoptosis. At 16 hours post-treatment with the apoptosis-inducing drugs actinomycin and etoposide, P2 cells were resuspended in binding buffer (BD Pharmingen) and stained with FITC-labeled Annexin V antibody (1 µg/ml) (BD Pharmingen). Discrimination between early apoptotic and late apoptotic or necrotic events was achieved by counterstaining cells with 5 µg/ml propidium iodide (PI) (BD Pharmingen). Cells were analyzed with FACSCalibur using CellQuest Pro or FlowJo software. For each sample 10,000 events were collected.

### PCR/3D-PCR

First round reaction parameters for human or quail *MT-COI* were 95 °C for 5 min, followed by 40 cycles (95 °C for 30 s, 60 °C for 30 s, and 72 °C for 2 min), and finally 10 min at 72 °C. Second round standard PCR and 3D-PCR^36^ were performed using the equivalent of 1 μl of the first round reaction as input. The second round for standard PCR was performed using a standard thermal cycler. The reaction parameters were 85 °C for 5 min, followed by 40 cycles (85 °C for 30 s, 60 °C for 30 s, and 72 °C for 2 min) and finally 10 min at 72 °C. Second round for 3D-PCR was performed using an Eppendorf gradient Mastercycler S. The reaction parameters were 81 to 87 °C for 5 min, followed by 40 cycles (81–87 °C for 30 s, 60 °C for 30 s, and 72 °C for 2 min), and finally 10 min at 72 °C. All amplifications were carried out using 2.5 U Taq (Bioline) DNA polymerase per reaction. PCR conditions and primers were described before^15,36^.

APOBEC3H cDNA from donors D1 and D2 were amplified with primers 3Hfor 5′ GAAACACGATGGCTCTGTTAACAGCC and 3Hrev, 5′ GGCAACTGACATGCCCCAGGG at 95 °C for 5 min, followed by 40 cycles (95 °C for 30 s, 60 °C for 30 s, and 72 °C for 2 min), and finally 10 min at 72 °C.

PCR and 3D-PCR products were purified from agarose gels (Nucleospin Extract II, Macherey-Nagel) and subsequently cloned using the TOPO TA Cloning kit (Invitrogen). All constructs were transformed and amplified in *E. coli* DH5α. Sequencing was outsourced to GATC.

### Real-time PCR

Total RNA was extracted from P2 cells, THP-1 and CD4^+^ T lymphocytes using RNeasy Plus Mini Kit (Qiagen) according to the manufacturer’s instructions. Synthesis of cDNA was performed with 1 µg RNA using the Quantitec Reverse Transcription Kit (Qiagen). Quantitative PCR was performed using cDNA samples and TaqMan Universal PCR Master Mix (Applied Biosystems) for human *A3A* to *A3H*. Primers and PCR conditions were previously described^56^. The data was normalized to the expression levels of the housekeeping reference gene *RPL13A*.

## Supplementary information

Supplementary Figure S1

Supplementary Figure S2
