# Supplementary material for: Genotoxic stress increases cytoplasmic mitochondrial DNA editing by human APOBEC3 mutator enzymes at a single cell level
Source: Sci Rep. 2019 Feb 28;9:3109. doi: 10.1038/s41598-019-39245-8 (PMC6395610; doi:10.1038/s41598-019-39245-8)
Supplement: Supplementary file 1 — Supplementary Figure S1 [file 41598_2019_39245_MOESM1_ESM.pdf]

	T	C	G	A
T		5	2	0
C	18		1	1
G	1	0		10
A	1	1	0	

D1 B05 @86.7°C
n=4712 bp

	T	C	G	A
T		12	3	5
C	784		1	3
G	3	1		0
A	4	1	6	

D1 F09 @86.1°C
n=6696 bp

	T	C	G	A
T		10	4	5
C	653		0	1
G	1	1		1
A	5	2	4	

D1 A06 @86.1°C
n=5704 bp

	T	C	G	A
T		6	1	1
C	21		1	1
G	0	1		9
A	0	0	1	

D2 G07 @86.7°C
n=4960 bp

	T	C	G	A
T		15	5	6
C	693		0	0
G	2	1		0
A	7	3	3	

D2 C08 @86.1°C
n=5952 bp

	T	C	G	A
T		9	4	4
C	741		1	2
G	4	1		0
A	3	1	5	

D2 E02 @86.1°C
n=6200 bp

Mutation matrices for hyperedited *MT-COI* DNA sequences in single $CD4^{+}$ T lymphocytes from donor D1 and D2 derived from cloned 3D-PCR products obtained at 86.7°C and 86.1°C. The numbers below the matrices (n) indicate the number of nucleotides analysed.

Genotoxic stress increases cytoplasmic mitochondrial DNA editing by human APOBEC3 mutator enzymes at a single cell level

Bianka Mussil, Rodolphe Suspène, Vincent Caval, Anne Durandy, Simon Wain-Hobson and Jean-Pierre Vartanian
