# Supplementary material for: Genotoxic stress increases cytoplasmic mitochondrial DNA editing by human APOBEC3 mutator enzymes at a single cell level
Source: Sci Rep. 2019 Feb 28;9:3109. doi: 10.1038/s41598-019-39245-8 (PMC6395610; doi:10.1038/s41598-019-39245-8)
Supplement: Supplementary file 2 — Supplementary Figure S2 [file 41598_2019_39245_MOESM2_ESM.pdf]

	T	C	G	A
T		7	0	1
C	15		2	0
G	0	1		12
A	1	0	1	

C08@86.7°C
n=5208 bp

	T	C	G	A
T		8	1	9
C	781		3	2
G	2	3		2
A	6	0	9	

A04+act D@86.1°C
n=7938 bp

	T	C	G	A
T		10	0	6
C	828		0	0
G	3	0		2
A	6	1	6	

F06+act D@86.1°C
n=13888 bp

	T	C	G	A
T		9	1	4
C	862		0	4
G	1	0		2
A	3	1	10	

D08+etop @86.1°C
n=6448 bp

	T	C	G	A
T		14	0	7
C	889		0	1
G	1	0		2
A	5	0	6	

E10+etop @86.1°C
n=14632 bp

Mutation matrices for hyperedited *MT-COI* DNA sequences after etoposide and actinomycin D treatments derived from cloned 3D-PCR products obtained at 86.7°C and 86.1°C. The numbers below the matrices (n) indicate the number of nucleotides analysed.

Genotoxic stress increases cytoplasmic mitochondrial DNA editing by human APOBEC3 mutator enzymes at a single cell level

Bianka Mussil, Rodolphe Suspène, Vincent Caval, Anne Durandy, Simon Wain-Hobson and Jean-Pierre Vartanian
